# Review of disability weight studies: comparison of methodological choices and values

**DOI:** 10.1186/s12963-014-0020-2

**Published:** 2014-08-23

**Authors:** Juanita A Haagsma, Suzanne Polinder, Alessandro Cassini, Edoardo Colzani, Arie H Havelaar

**Affiliations:** 1 Department of Public Health, Erasmus Medical Center, Rotterdam, 3000 CA, The Netherlands; 2 Office of the Chief Scientist, European Centre for Disease Prevention and Control, Stockholm, SE-171 83, Sweden; 3 National Institute for Public Health and the Environment, Laboratory for Zoonoses and Environmental Microbiology, Bilthoven, 3720 BA, The Netherlands; 4 Utrecht University, Institute for Risk Assessment Sciences, Utrecht, 3508 TD, the Netherlands

**Keywords:** Value of life, Disease burden, Disability adjusted life years, Summary measure of population health, Prioritisation

## Abstract

**Introduction:**

The disability-adjusted life year (DALY) is widely used to assess the burden of different health problems and risk factors. The disability weight, a value anchored between 0 (perfect health) and 1 (equivalent to death), is necessary to estimate the disability component (years lived with disability, YLDs) of the DALY. After publication of the ground-breaking Global Burden of Disease (GBD) 1996, alternative sets of disability weights have been developed over the past 16 years, each using different approaches with regards to the panel, health state description, and valuation methods. The objective of this study was to review all studies that developed disability weights and to critically assess the methodological design choices (health state and time description, panel composition, and valuation method). Furthermore, disability weights of eight specific conditions were compared.

**Methods:**

Disability weights studies (1990¿2012) in international peer-reviewed journals and grey literature were identified with main inclusion criteria being that the study assessed DALY disability weights for several conditions or a specific group of illnesses. Studies were collated by design and methods and evaluation of results.

**Results:**

Twenty-two studies met the inclusion criteria of our review. There is considerable variation in methods used to derive disability weights, although most studies used a disease-specific description of the health state, a panel that consisted of medical experts, and nonpreference-based valuation method to assess the values for the majority of the disability weights. Comparisons of disability weights across 15 specific disease and injury groups showed that the subdivision of a disease into separate health states (stages) differed markedly across studies. Additionally, weights for similar health states differed, particularly in the case of mild diseases, for which the disability weight differed by a factor of two or more.

**Conclusions:**

In terms of comparability of the resulting YLDs, the global use of the same set of disability weights has advantages, though practical constraints and intercultural differences should be taken into account into such a set.

## Introduction

Human health is threatened by an array of diseases and injuries. Limited resources compel policymakers to focus on threats that are most relevant in terms of public health. An objective tool that aids policymakers in setting priorities in resource allocation is the disability-adjusted life year (DALY). The DALY measures the burden of disease, i.e., it aggregates the total health loss at population level into a single index by summarizing a) years of life lost due to premature death (YLLs) and b) years lived with disability (YLDs) [1]. In this way the DALY estimations allow comparability between the impact of diseases and provide knowledge on the size of health problems and the potential benefit of proposed measures set against similar and comparable data of other health problems [2],[3].

An essential factor for establishing YLDs is the disability weight, a value assigned to living with disability. This value, anchored between 0 (perfect health) and 1 (equivalent to death), reflects the impact of a specific health condition. The values of the disability weights are commonly based on preferences obtained from a panel of judges [4]. Preferences are defined as quantitative expressions or valuations for certain health states, which reflect the relative desirability of a health state [5],[6]. Empirical research has shown that preferences are dependent on the composition of the panel, with patients valuing their disease as less unfavorable compared to the general public [7]-[9], though these findings have been disputed [10],[11]. Other methodological aspects that may influence preferences for a certain health state are the way the health state and duration of the health state are described and the valuation method that is used. Each of these aspects affect the preferences that are measured, which in turn affect the values of the disability weights [12].

For the ground-breaking Global Burden of Disease (GBD) 1996 study that estimated the total burden of disease worldwide a large set of global disability weights was derived [1],[13]. However, because of a need to validate and improve the novel valuation procedure, a need for disability weights that reflected preferences of the national population and/or because of practical limitations of the GBD 1996 disability weight (i.e., lack of disability weights for certain diseases or lack of differentiation between different health states within one disease or disease group), alternative sets of disability weights have been developed over the past 16 years, each using different approaches with regards to the panel, health state description, and valuation methods.

This review aims to provide an overview of all studies that developed disability weights and to compare the methodological design choices. Four key choices were addressed: (1) the health state description, (2) time presentation, (3) panel composition, and (4) the valuation method. Furthermore, disability weights for 15 specific disease and injury groups resulting from the disability weight studies were compared with the aim to assess the influence of the description of the health condition and other design choices on difference in the disability weights.

## Review

### Disability weights ¿ design choices

**Figure 1:**
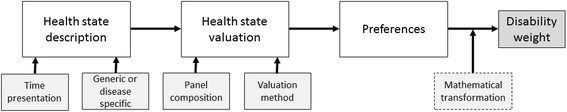
Conceptual model of assessing disability weights and its design choices.

Figure 1 shows a conceptual model of assessing disability weights and its four main design choices. The first choice is the health state description. The choices here are to describe the disease in generic terms or in disease-specific terms. A disease-specific description depicts the disease label and/or clinical description; it indicates the cause and/or the specific health effects of the condition. A generic health state description depicts the functional health independent of the actual underlying condition. For this purpose a multi-attribute utility instrument (MAUI) is used [14]. With MAUI, generic attributes are used to classify health states [7],[15],[16]. Firstly, patients describe their health state by choosing a functional level for each attribute. Using weights for the separate attributes, the reported functional level on the attributes is then converted into a summary score which fits within the 0¿1 range, where 1 is perfect health (the reverse direction compared to DALY weights). The weights that are used to convert the health states into a disability weight are derived at an earlier stage and are based on preference data of the general population for health states described with the generic attributes. This approach is similar to the approach that is used to derive quality-adjusted life year (QALY) weights, except that one extra step is taken to transform QALY weights into disability weights. Widely used MAUIs include the EQ-5D health questionnaire and Health Utilities Index (HUI) [17],[18]. For the EQ-5D several tariffs exist for calculating EQ-5D summary scores. Two other ways to derive health state valuations using the EQ-5D are 1) to use the visual analog scale (VAS) that accompanies the EQ-5D and 2) to use the health description system of the EQ-5D to describe a health state, either with our without additional disease information, which is then submitted to a panel of experts or lay people to derive disability weights [19],[20].

The second design choice concerns the time presentation. The time presentation of the health state can be distinguished into period profiles and annual health profiles. With period profiles, the underlying assumption is that that the value of the health state is not affected by the duration of the health state [21],[22]. With the annual profile approach, the course of the health state ¿ the disability profile ¿ is described over a period of one year [4],[23]. This allows valuation of conditions with an acute onset, conditions with a short duration, episodic diseases such as epilepsy, and conditions that are characterized by complex and heterogeneous recovery patterns. An example of an annual profile health state description is a person who has gastroenteritis for a period of seven days but for the remainder of the year the person is healthy.

The third choice is the panel composition. The panel providing the preferences may consist of patients or valid proxies, medical experts, or members of the general public.

The fourth main design choice concerns the valuation method. To measure individual preferences, several valuation methods exist. These valuation methods include pairwise comparison, the VAS, time trade-off (TTO), person trade-off (PTO), and standard gamble (SG). Each of these valuation methods has different properties that affect the preferences that are measured. The TTO, PTO, and SG are choice-based valuation methods; asking to make trade-offs in time (TTO), person-years (PTO), or risk of death against improvement in health. For a detailed overview of these valuation methods see [24].

### Literature review - selection criteria and definitions

This review is restricted to studies that assessed disability weights for burden of disease measurements, expressed in DALY estimates. Empirical studies in the international peer-reviewed journals and grey literature published in English in the period 1990 to 2012 were included. Studies in established market economies and low- and middle-income countries were all included. This review included studies that derived disability weights for several groups of health outcomes or a specific group of illnesses (for instance: periodontal disease or cancer). We excluded studies that derived a disability weight for one single health state (because these studies do not give information about the relative desirability of a health state compared to other health states), studies that derived disability weights for risk factors (such as environmental factors, e.g., noise), and studies that derived severity weights for QALYs.

### Literature review - data sources and search strategy

Searches of eligible studies were conducted in Medline (PubMed) and EMBASE. All international peer reviewed articles published in the period between January 1, 1990 and December 31, 2012 were included in the searches. Searches for eligible grey literature were conducted in Google Scholar. Search terms used for general burden of disease studies were: ¿disability weight¿, ¿severity weight¿, ¿burden of disease¿, ¿disability adjusted life year¿, ¿disability-adjusted life year¿, ¿DALY¿. Keywords were matched to database-specific indexing terms. In addition to database searches, reference lists of review studies and articles included in the review were screened for titles that included key terms.

### Literature review - data extraction

Relevant papers were selected by screening the titles (first step), abstracts (second step), and entire articles (third step) retrieved through the database searches. During each step, respectively, the title, abstract, or entire article was screened to ensure that it met the selection criteria listed above. This screening was conducted independently by two researchers (JH and SP). Disagreement about eligibility between the reviewers was resolved through discussion.

Selected full articles were critically appraised by two reviewers (JH and SP), using data extraction forms, which included information on the study population, details regarding the methods used to calculate YLL and YLD, main conclusions, etc. Their reports were compared and disagreements were resolved by discussion.

### Comparison of disability weights

Disability weights of 15 specific diseases/injuries were compared. We selected 15 diseases/injuries that represent the complete spectrum of severity (from mild conditions through very severe conditions) that were included in more than one disability weight study. Eleven of these health states were selected from the 22 indicator conditions of the GBD 1996 study. The four other conditions were selected because one or more of the disability weights studies focused on this single cause of disease (e.g., periodontal disease, stroke, or depression).

## Results

**Figure 2:**
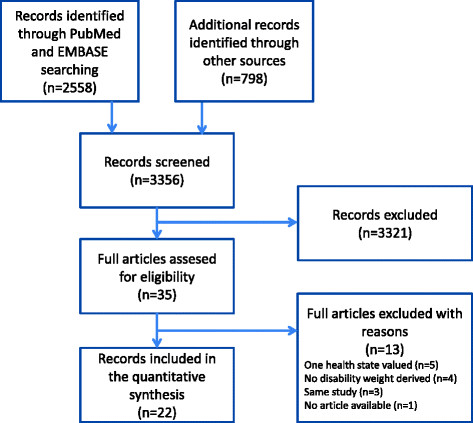
Flow diagram of the search of existing burden of disease studies.

Figure 2 shows the flow diagram of the search of existing burden of disease studies and the main reasons for exclusion. In total, 22 disability weights studies were included. Table 1 presents a detailed overview of the general information, health states that were valued, and methodological design choices of each of the 22 studies. Three studies were global disability weights studies [25]-[27] and one study included a panel of judges from four countries (United States, South Korea, China, and Taiwan; [28]). All other studies concerned particular countries or regions.

**Table 1:** Included studies: Panel of judges, health state description, and time presentation

**Year**	**Study**	**Ref no.**	**Region**	**Multiple or single cause?**	**Panel composition**	**N panel**	**N health states**	**Health state description**	**Time presentation**	**Valuation methods** ***(% of total number of health states valued by each of the methods)***
1996	Murray et al.	[25]	Global	M	ME	10	483	DS	PP	<1% PTO, 99% VAS
1997	Stouthard et al.	[19]	Netherlands	M	ME	38	175	DS?+?EQ-5D	PP & AP	10% PTO, 90% VAS
1999	Üstün et al.	[26]	Global	M	HP, PM, PT, PX	241	17	DS	PP	100% ranking
2000	Havelaar et al.	[29]	Netherlands	M	ME	35	NA	NA	PP	Interpolation
2000	Jelsma et al.	[30]	Zimbabwe	M	PP, ME	68	22	NA	PP	VAS (ranking)
2000	Mathers et al.	[31]	Australia	M	Model	-	Model	MAUI (EQ-6D)?+?DDW	PP	EQ-6D: Not applicable
2002	Baltussen et al.	[32]	Burkina Faso	M	PP, HP	56	9	DS (scenarios)	PP	Adapted VAS/interpolation
2003	Schwarzinger et al.	[33]	Europe	M	ME, NHP	232	15	DS?+?EQ-5D	PP	100% VAS, 60% TTO, 60% PTO
2004	Brennan et al.	[34]	Australia	S (oral)	Model	-	18	MAUI (EQ-5D)	PP	EQ-6D: Not applicable
2005	Kruijshaar et al.	[35]	Netherlands	M	ME	49	9	DS?+?EQ-5D	PP	100% interpolation
2007	Brennan et al.	[36]	Australia	S (oral)	Model	-	6	MAUI (EQ-5D)	PP	EQ-6D: not applicable
2007	Yoon et al.	[37]	Korea	M	ME	30	123	NA	PP	13% PTO, 87% interpolation
2008	Basiri et al.	[38]	Iran	S (urologic)	ME	10	76	NA	PP	100% interpolation
2008	Haagsma et al.	[20]	Netherlands	S (injury)	PP	143	44	DS?+?EQ-5D	AP	100% VAS, 100% TTO
2008	Haagsma et al.	[39]	Netherlands	M	PP	107	39	DS?+?EQ-5D	AP	100% VAS, 100% PTO
2009	Haagsma et al.	[23]	Netherlands	S (injury)	Model	-	7	MAUI (EQ-5D)	PP	EQ-5D: not applicable
2009	Hong et al.	[28]	US/Southeast Asia	S (stroke)	ME	9	5	NA	NA	100% PTO
2009	Lai et al.	[40]	Estonia	M	ME	25	283	DS	PP	9% PTO, 91% VAS
2010	Kwong et al.	[41]	Canada	M	Model	-	Model	MAUI (CLAMES)	PP	CLAMES: not applicable
2011	Lyons et al.	[42]	UK	S (injury)	Model	-	13	MAUI (EQ-5D)	PP	EQ-5D: not applicable
2012	Salomon et al.	[27]	Global	M	PP	30230	220	DS (without label)	PP	100% Paired comparison
2011	Van Spijker et al.	[43]	Netherlands	M	ME	16	12	DS?+?EQ-5D	PP	100% VAS

NA =?not available.

Multiple or single cause of disease: M =?multiple, S =?single.

Panel: ME =?medical experts, HP =?health professionals, PM =?policymakers, PT =?patients/people with disabilities, PX =?patient proxies, PP =?population.

Health state description: DS =?disease-specific, MAUI =?generic multi-attribute utility.

EQ-5D =?multi-attribute utility instrument that consists of five attributes (mobility, self-care, usual activities, pain/discomfort. and anxiety/depression).

EQ-6D =?EQ-5D appended with a cognitive attribute.

Clames model =?Classification and Measurement System of Functional Health; a combination of HUI, SF36, and EQ-5D attributes.

Time presentation: PP =?period profile, AP =?annual profile.

Valuation method: VAS =?visual analog scale, TTO =?time trade-off, PTO =?person trade-off.

The majority of the 22 disability weights studies developed disability weights for a variety of illnesses. Eight studies concerned disability weights for a specific category (i.e., oral/periodontal diseases [34],[36], infectious diseases [29], injuries [20],[23],[42], urological diseases [38], or stroke [28]. The total number of health states that were valued varied widely from five [28] to 483 [25].

### Methodological design choices to render the disability weights

#### Health state description

Five studies (23%) used a MAUI model to assess disability weights for health states [23],[34],[36],[42],[44]. Four of these studies used the EQ-5D model or EQ-6D model (also known as the EQ-5D?+?model; this model includes an additional cognitive domain) [23],[34],[36],[42]. One study developed a new health status classification system, namely the classification and measurement system of functional health (CLAMES), which combines selected attributes of several MAUIs [44].

Furthermore, Mathers et al. used a regression model based on the Dutch Disability Weights (DDW) study to derive disability weights for diseases not included in that study and to adjust annualized estimates for duration for acute conditions [31].

Eleven studies (50%) depicted the health states in a disease-specific way [19],[20],[25]-[27],[30],[32],[33],[35],[39],[43]. These disease-specific health state descriptions consisted of short descriptions or disability scenarios with illustrations or descriptions that included a disease-specific description of symptoms and generic information. Five studies did not report how the health states were depicted that were valued [28]-[30],[37],[40].

#### Time presentation

All studies presented the health states as period profiles, apart from three Dutch disability weights studies, which used the annual profile approach [19],[20],[39]. The annual profile disability weights for short-term diseases are much lower compared to period profile disability weights.

#### Panel composition

Of the 17 studies that did not use a MAUI, 59% (n =?10) asked medical experts or health professionals to value health states [19],[25],[28],[29],[33],[35],[37],[38],[40],[43]. Three studies derived preferences from a population panel [20],[27],[39]. Two studies included two panels: medical experts and people from the population [30],[32]. Both studies showed differences between disability weights derived from these two groups. Jelsma et al. report a correlation of 0.32 (p =?0.153) between the ranking of health professionals and people from the population. Baltussen et al. showed that medical experts valued five of the nine health states significantly lower compared to people from the population. Üstün et al. derived preferences from health professionals, policymakers, and people with disabilities and their carers [26] and found that the average correlation of rank orders between different informant groups was 0.76.

The number of judges varied from nine [28] to 30,230 [27].

#### Valuation method

Of the non-MAUI studies, nine studies (53%) derived preferences using a two-step procedure [19],[25],[29],[32],[33],[35],[37],[38],[40]. Firstly, preferences for a small subset of health states were derived using a trade-off method (PTO or TTO). The second step consisted of an interpolation exercise, where the panel of judges was asked to interpolate the remaining health states using the values for the subset. Other studies used only ranking [26],[30], pairwise comparison with additional information on population health equivalence [27], or VAS [43] to derive valuations. In three studies, all health states were evaluated with a trade-off method [20],[28],[39]. Comparison of studies that used more than one valuation technique showed that agreement of rankings with VAS were slightly higher compared to agreement of rankings with TTO/PTO [19],[20],[33],[39].

### Experimental design

Table 2 presents an overview of the experimental design of the 22 studies. Apart from the GBD 2010 disability weights study [27], the disability weights were derived using a written questionnaire. Some studies arranged a panel meeting with group discussion during which preferences were derived, whereas other studies used individual questionnaires. Also, a combination of panel meetings and individual questionnaires were used.

**Table 2:** Experimental design used to render the disability weights

**Experimental design**	**Number of studies**	**References**
Panel meeting/focus group discussion	7	[25],[29],[30],[33],[37],[40],[45]
Questionnaire	4	[27],[35],[38],[43]
Panel meeting?+?questionnaire	3	[19],[20],[39]
Questionnaire?+?panel meeting	1	[32]
Questionnaire?+?panel meeting?+?questionnaire	1	[28]
Interview	2	[26],[27]

### Comparison of disability weights

Comparisons of disability weights for 15 specific disease and injury groups shows that there is large variation in disease stages that are used in the studies (Table 3). For instance, the number of health states of CVA/stroke ranges from one [40] to five [27],[28]. Also, there is large variation between studies in the values of the disability weights for similar health states. For instance, the disability weight for paraplegia ranges from 0.047 (treated paraplegia) and 0.440 (untreated paraplegia) [27] to 0.725 [25], and the disability weight for severe depression ranges from 0.147 [40] to 0.83 [19]. Particularly in the case of mild health states, the disability weight can differ by a factor of two or more. The disability weight for cystitis ranges from 0.01 [19] to 0.023 [41]. For severe gastroenteritis, the disability weights (period profile) range from 0.061 [27] to 0.393 [29].

**Table 3:** Disability weight of eight diseases/injuries

	**Author(s) of the study**	**Chronic sequelae of CVA/stroke**	**Paraplegia**	**Dental caries**	**Rheumatoid arthritis**	**Diabetes**	**Depression**	**Gastro-enteritis**	**Acute cystitis**
[25]	Murray et al., 1996	Untreated 0.282 (varies with age between 0.262 - 0.301); treated 0.241 (varies with age between 0.224-0.258)	0.725	0.081	Untreated 0.233, treated 0.174	Treated 0.012; untreated 0.033	Treated 0.302; Untreated 0.600	0.105 (varies with age between 0.086 - 0.119)	-
[19],[46]	Stouthard et al., 1997	Mild impairments 0.36 (CI 0.23- 0.49); Moderate impairments 0.63 (CI 0.543-0.718); Severe impairments 0.92 (CI 0.853-0.994)	0.57 (CI 0.489-0.651)	0.01 (CI 0.001-0.009)	Mild 0.21 (CI 0.127-0.303); moderate 0.37 (CI 0.219-0.515); severe 0.94 (CI 0.92-0.961)	Uncomplicated 0.07 (CI 0.047-0.094; with neuropathy 0.19 (CI 0.126-0.255); with nephropathy 0.29 (CI 0.201-0.38)	Mild 0.14 (CI 0.086-0.194); moderate 0.35 (CI 0.272-0.425); severe 0.76 (CI 0.556-0.971); severe with psychotic features 0.83 (CI 0.748-0.916)	Uncomplicated course 0.01 (CI 0.001-0.009); complicated course 0.03 (CI 0.018-0.039)	0.01 (CI 0¿0.039)
[26]	Üstün et al., 1999	-	r	-	r		r	-	-
[29]	Havelaar et al., 2000	-	-	-	-		-	Severe gastroenteritis 0.393 (CI 0.049-0.821)	
[30]	Jelsma et al., 2000	-	r	-	r	-	r	-	-
[31],[47]	Mathers et al., 2000	-	-	Episode resulting in tooth loss 0.014	-	-	-	-	-
[32]	Baltussen et al., 2002	-	PP: 0.42; HP: 0.55	-	-	PP: 0.49; HP: 0.34	PP: 0.74; HP: 0.66	-	
[33]	Schwarzinger et al., 2003	Moderate impairments 0.68	-	-	-	Uncomplicated 0.34	Severe 0.78	-	
[34]	Brennan et al., 2004	-	-	0.044 (0.013-0.076)	-	-	-	-	-
[35]	Kruijshaar et al., 2005		-	-	-		Mild 0.19 (CI 0.16-0.22); moderate 0.51 (CI 0.46-0.55); severe 0.84 (0.80-0.88)	-	
[36]	Brennan et al., 2007	-	-	-	-	-	-	-	-
[37]	Yoon et al., 2007	NA	NA	NA	NA	NA	NA	NA	NA
[38]	Basiri et al., 2008	-	-	-	-	-	-	-	0.018 (?0.047-0.083)
[20]	Haagsma et al., 2008	-	Acute 0.563 (0.495-0.631), stable 0.656 (0.525-0.786)	-	-	-	-	-	-
[39]	Haagsma et al., 2008	-	0.551 (0.36-0.74)	-	-	-	-	-	-
[23]	Haagsma et al., 2009	-	-	-	-	-	-	Mild 0.010 (?0.005-0.025), moderate 0.015 (0.005-0.025), severe 0.041 (0.014-0.067)	-
[28]	Hong et al., 2010	mRS1 0.046 (CI 0.004-0.088); mRS2 0.212 (0.175-0.250); mRS3 0.331 (0.292-0.371); mRS4 0.652 (0.625-0.678); mRS5 0.944 (0.873-1.015)	-	-	-	-	-	-	-
[40]	Lai et al., 2009	0.547	0.669	0.078	0.203	Insulin dependent 0.264, insulin non-dependent 0.029	0.147	0.011	
[41],[44]	Kwong et al., 2010	-	-	-	Active episode 0.262; chronic active 0.058; advanced damage 0.465	-	Mild 0.122; moderate 0.440; severe 0.558	Mild 0.023; moderate 0.041; severe 0.086	0.023
[42]	Lyons et al., 2011	-	-	-	-	-	-	-	-
[27]	Salomon et al., 2012	mild: 0.021 (0.011-0.037)	untreated 0.440 (CI 0.290-0.588), treated	Symptomatic: 0.012 (0.005-0.023)	Legs, mild: 0.023 (0.013-0.039)	-	Mild: 0.159 (UI 0.107-0.223)	Mild: 0.061(0.036-0.093)	-
		moderate: 0.076 (0.050-0.110)							
			0.047 (CI 0.029-0.072)		Legs, moderate: 0.079 (0.053-0.115)		Moderate: 0.406 (UI 0.276-0.551)	Moderate: 0.202 (0.133-0.299)	
		moderate plus cognition problems: 0.312 (0.211-0.433)							
					Legs, severe: 0.171 (0.117-0.240)		Severe: 0.655 (UI 0.469-0.816)	Severe: 0.281 (0.184-0.399)	
		severe: 0.539 (0.363-0.705)							
					Arms, mild: 0.024 (0.014-0.041)				
					Arms, moderate: 0.114 (0.077-0.159)				
					Arms, severe: 0.292 (0.077-0.159)				
					Generalised, moderate: 0.292 (0.197-0.410)				
					Generalised severe: 0.606 (0.42-0.77)				
		severe plus cognitive problems: 0.567 (0.394-0.738)							
[43]	Van Spijker et al., 2011	-	-	-	0.33 (CI 0.05-0.61)	-	Severe with psychotic features 0.74 (CI 0.40-1.08)	-	-
**Ref nr.**	**Author(s) of the study**	**Severe anemia**	**Infertility**	**Below the knee amputation**	**Deafness**	**Mild mental retardation**	**Dementia**	**Blindness**	
[25]	Murray et al., 1996	0.087-0.093 (varies with age)	0.180	0.3	Untreated 0.213-0.233; treated 0.168-0.175	Untreated 0.469-0.485; treated 0.394-0.468	Untreated 0.600; treated 0.302	Untreated 0.600; treated 0.488-0.493	
[19],[46]	Stouthard et al.,1997		0.11 (CI 0.03-0.20)		Childhood 0.23 (0.12-0.33); elderly 0.37 (0.34-0.41)	0.29 (CI 0.09-0.50)	Mild 0.27 (CI 0.13-0.42); moderate 0.63 (CI 0.41-0.86); severe 0.94 (CI 0.93-0.95)	0.43 (CI 0.34-0.52)	
[26]	Üstün et al., 1999	r	r	r	r	r	r	r	
[29]	Havelaar et al., 2000	-	-	-	-	-	-	-	
[30]	Jelsma et al., 2000	r	r	r	r	r	r	r	
[31],[47]	Mathers et al., 2000		-	-	-	-	-	-	
[32]	Baltussen et al., 2002	-	-	-	PP: 0.16; HP: 0.11	-	-	PP 0.35; HP 0.36	
[33]	Schwarzinger et al., 2003	-	-	-	-	-	Mild dementia 0.46	-	
[34]	Brennan et al., 2004	-	-	-	-	-	-	-	
[35]	Kruijshaar et al., 2005	-	-	-	-	-	-	-	
[36]	Brennan et al., 2007	-	-	-	-	-	-	-	
[37]	Yoon et al., 2007	NA	NA	NA	NA	NA	NA	NA	
[38]	Basiri et al., 2008	-	-	-	-	-	-	-	
[20]	Haagsma et al., 2008	-	-	-	-	-	-	-	
[39]	Haagsma et al., 2008	-	-	-	-	-	-	-	
[23]	Haagsma et al., 2009	-	-	-	-	-	-	-	
[28]	Hong et al., 2010	-	-	-	-	-	-	-	
[40]	Lai et al., 2009	0.168	0.547	0.747	0.254	0.242	0.261	0.478	
[41],[44]	Kwong et al., 2010		-			0.122		Diabetic retinopathy 0.248	
[42]	Lyons et al., 2011	-	-	-	-	-	-	-	
[27]	Salomon et al., 2012	0.164 (UI 0.112-0.228)	Primary 0.011 (UI 0.005-0.021); secondary 0.006 (UI 0.002-0.013)	Untreated 0.164 (UI 0.111-0.229); treated 0.021 (UI 0.011-0.035)	Complete 0.033 (UI 0.020-0.052); complete with ringing 0.092 (UI 0.061-0.134)	0.031 (UI 0.018-0.049)	Mild 0.082 (CI 0.055-0.117); moderate 0.346 (CI 0.233-0.475); severe 0.438 (CI 0.299-0.584)	0.195 (0.132-0.272)	
[43]	Van Spijker et al., 2011	-	-	-	-	-	-	-	

NA=not available, r=ranking, no actual value was established, CI=95% confidence interval if reported, UI=Uncertainty interval if reported.

For four studies, we have tabulated the rankings of 12 diseases and calculated the rank order correlation (?) for each of the sets of disability weights. The results shown in Table 4 reveal that there is consistency in the rankings between the GBD 1996, the DDW, and the Estonian disability weights study, with ? ranging between 0.426 (p <?0.05) and 0.626 (p <?0.01). However, the rank order correlations showed a lack of consistency in the rankings of the GBD 2010 study and the other three disability weights studies included in the comparison.

**Table 4:** Rank order correlations between four disability weights studies

	**Murray et al., 1996**	**Stouthard et al., 1997**	**Lai et al., 2009**	**Salomon et al., 2013**
Murray et al., 1996	X	0.462*	0.626**	0.351
Stouthard et al., 1997		X	0.534*	0.107
Lai et al., 2009			X	0.515*
Salomon et al., 2013				X

**p <?0.01; *p <?0.05.

It seems that studies that used ranking and VAS and studies that provided a short disease-specific health state description resulted in slightly worse disability weights compared to studies that presented generic information on functional health in addition to the disease-specific information (Table 3). However, the actual descriptions of each of the selected conditions were not available. Therefore, it was not possible to perform a detailed analysis to assess whether the differences are related to the presentation of the health state and/or other methodological design choices.

## Discussion

Twenty-two disability weights studies were included in the review. The total number of health states valued in these studies varied from five to more than 400. The results of this systematic review showed that there is variation in methods used to derive disability weights. However, most studies used a disease-specific description of the health state, a panel that consisted of medical experts, and a nonpreference-based valuation method to assess the values for the majority of the disability weights. Comparisons of disability weights across 15 specific disease and injury groups showed that the subdivision of a disease into separate health states (stages) differed markedly. Additionally, weights for similar health states differed, particularly in the case of mild diseases, for which the disability weight differed by a factor of two or more.

### Coverage of diseases and disease staging

As mentioned above, we found marked differences in coverage of diseases and subdivision of a disease or injury into different health states. The GBD 1996 and the Estonian disability weights sets cover a wider range of conditions than the Dutch disability weights, but are generally less specific in terms of the specific disease stages to which they refer. The set of Dutch disability weights covers a restricted range of conditions compared to the GBD disability weights, but it provides more detailed differentiation between disease stages and severities, thus allowing more detailed disease models in estimating the YLDs than is possible with the GBD or Estonian disability weights [40].

Disability weights studies that focused on a single cause of disease (e.g., periodontal disease, stroke, or depression) also included more detailed disease stages. Often these studies were conducted because the disability weights that are available from the GBD 1996 set are not tailored to the available data on incidence or prevalence. If, for example, the impact of the disease among incident cases is markedly better or worse than that represented by the available ¿disability weights¿, data on the incidence of a disease cannot be accurately linked to the functional outcomes of that disease. In addition, disability weights for certain health outcomes may not be available or appropriate, e.g., for infectious diseases (Guillain-Barré syndrome, irritable bowel syndrome, reactive arthritis) [39], injuries (concussion) [20], or periodontal disease [34],[36].

### Panel composition and contextual differences

Using disability weights based on societal preferences has been recommended, because burden of disease studies are primarily used as a tool for guiding decision-making on resource allocation at the population level [3]. Nonetheless, the majority of studies asked medical experts or health professionals to value health states. Three studies asked both medical experts and members of the general public [26],[30],[32]. The results of two of these studies showed significant differences between disability weights derived from these two groups. Also, it should be noted that diseases and injuries rated as less severe by experts in a high-income country might be rated as more burdensome by people in health care in low-income settings. Two studies compared health state valuations among residents of several countries. A study among European countries showed that ranking of health states is similar across countries [33]. However, Üstün et al. found that for the majority of health conditions there were significant differences in ranking between 14 countries. Salomon et al., on the other hand, reported a high degree of consistency between sites, with the exception of one site [27]. Previous studies have shown that there are clear contextual differences in the ways people perceive health problems and how such problems affect their lives [48]-[52]. The findings from Jelsma et al. suggest that contextual differences may be stronger among lay people compared to health professionals [30]. Further research is needed to gain greater insight into the effect of contextual differences on disability weights.

### Health state description

Five studies used a MAUI model (e.g., EQ-5D or CLAMES) to assess disability weights. A MAUI model describes the health state with generic attributes only, whereas disease-specific health state descriptions may include disability scenarios with illustrations, a specific description of the symptoms, and/or information on treatment. Disease-specific health state descriptions have been shown to be more sensitive for the detection and quantification of small changes [53], see e.g. Stouthard et al. [46] who combined disease-specific information with EQ-5D data [46]. They found that low back pain and prostate cancer, health states with a similar EQ-5D profile but a different disease label, yielded different values. This indicates that disease-specific health state descriptions provide information that is not reflected in the generic health states but which matters for health state valuation. Which information is reflected in the generic health state, however, depends on the MAUI that is used, as they differ in the number and type of health domains that are included, the total number of possible health states, and the health state valuation techniques that are used to assess the weights that are assigned to the health domains. When an extensive disease-specific description of a health state is used, it is important to realize that the description may produce information bias because of message-framing effects [54]. Empirical studies have shown that the manner of depicting the health state affects preferences for these health states [55]. Secondly, including information on the symptoms of the disease, the impact on daily functioning, and duration in the description of the health state may result in cognitive overload of the panel of judges, especially if the panel of judges consists of members of the general public.

We recommend using a combination of a generic and disease-specific health state, because it has been shown that even for expert panels, adding a generic description of functional health status to the diagnostic disease label is necessary to standardize the stimulus [33],[46],[56]. Furthermore, utilizing an unadjusted MAUI model, such as the EQ-5D, to assess disability weights may be inconsistent with the objective of DALYs, because disability weights in GBD and other population-based burden of disease studies are adjusted to ascertain that the burden of disease from all causes adds up to the total burden of disease in the population. The unadjusted MAUI-based disability weights do not have such a restriction.

### Time presentation: period versus annual profiles

Disability weights can be subdivided into period profile and annual profile disability weights. Period profile disability weights assume independence between duration and disability and they require that the health state remains constant over time. This assumption is untenable for disorders that are characterized by a complex time-severity course [57], because it is impossible to disaggregate complex time patterns into a limited number of homogeneous stages. A solution proposed to overcome this problem was the annual profile approach, which describes the health profile over a period of one year [4]. The results of this systematic review showed that the annual profile approach has not been adopted internationally. This may be due to several reasons. Firstly, it has been argued that the annual profile approach may overvalue diseases with a mild and rapid course [58], though this may be corrected by using a relevance criterion for the inclusion of incident or prevalent cases [39]. A second, more practical, reason may be that the annual profile disability weights are rather inflexible with regard to time. If health states with different durations than the duration included in the annual profile are needed, they cannot be derived by back-calculation. Thus, the initial disability weights have to be applied, even though the duration does not match, or a new annual profile disability weight has to be derived with a new panel study.

### Valuation methods

A majority of disability weight studies used ranking, interpolation, paired comparison, or the VAS to assess the values for disability weights. These valuation methods lack the trade-off feature; they do not ask to sacrifice something valuable in order to assess the undesirability of the health state. Therefore, in a technical sense, these studies do not assess preferences but values [14]. The values elicited with VAS, and to a lesser degree with paired comparison, ranking, and interpolation, give information about the relative desirability of a health state compared to other health states (A is valued higher than B in a VAS, therefore A is preferred to B), but it is impossible to reasonably infer the trade-offs that people are willing to make [6]. Regarding the actual values of the disability weights, many studies have found that health state valuations with the VAS tend to be higher compared to equivalent valuations with choice-based valuation methods [20],[59],[60]. The results from this study appear to support those findings.

### Validity

In the absence of a gold standard for the disability component it is difficult to evaluate the validity of disability weights. One way to study the validity of disability weights is by tabulating and comparing the rankings of matching diseases and injuries of several studies. These comparisons showed a high degree of consistency in ranking between the GBD 1996 study, DDW, the Estonian disability weights study, and the GBD 2010 study. However, it should be noted that to calculate a disability weight for the 12 matching diseases and injuries we needed to aggregate disability weights for different disease stages of several studies very crudely. A second approach to assess validity is to compare the disability weights of disease stages within a certain disease or injury. Many studies subdivided a disease or injury into disease stages and comparison of the disability weights of these disease stages showed that the more severe health states have a higher disability weight compared to less severe health states, indicating high face validity.

### Comparison of disability weights

A major finding of this study is the fact that the values of the disability weights across studies differ markedly. As a result, disability weights from studies with different designs cannot be used interchangeably. This raises the question of whether DALY estimates from studies using different sets of disability weights can be compared to each other, since a disability weight that is twice as high might result in a YLD that is twice as high if the same duration and incidence or prevalence numbers are applied. The GBD 2010 study showed major shifts in rankings of causes of burden of disease, and these differences can, to some extent, be traced back to marked differences in disability weights (e.g., back pain and sensory disability) [61]. Hence, to meet the purpose of the DALY, namely to assess the burden of disease and injury at the population level for comparability of impacts of different diseases and risk factors over time and between regions, it is important to use the same set of universal disability weights.

## Conclusions

Methodological constraints, contextual differences, and practical limitations have urged burden of disease researchers to derive alternative sets of disability weights, each using a different approach with regard to the panel, health state description, and valuation methods, resulting in widely varying values for similar health states. However, in terms of comparability of the resulting YLDs, the global use of the same set of disability weights is preferable.

## Competing interest

JH, SP, and AH: No competing interests.

AC and EC work for the European Centre for Infectious Disease Prevention and Control (ECDC) that funded this study.

## Authors¿ contributions

JH, SP, AC, EC, and AH developed the study concept and design. JH and SP performed the literature search and data extractions. JH, SP, and AH analyzed and interpreted the data. JH and SP drafted the manuscript. JH, SP, AC, EC, and AH critically revised the manuscript for important intellectual content. All authors read and approved the final manuscript.
